# Divergent neuroimmune signatures in the cerebrospinal fluid predict differential gender-specific survival among patients with HIV-associated cryptococcal meningitis

**DOI:** 10.3389/fimmu.2023.1275443

**Published:** 2023-12-13

**Authors:** Samuel Okurut, David R. Boulware, Elizabeth Okafor, Joshua Rhein, Henry Kajumbula, Bernard S. Bagaya, Freddie Bwanga, Joseph O. Olobo, Yukari C. Manabe, David B. Meya, Edward N. Janoff

**Affiliations:** ^1^ Translation Sciences Laboratory, Research Department, Infectious Diseases Institute, Makerere University, Kampala, Uganda; ^2^ Department of Medical Microbiology, School of Biomedical Sciences, College of Health Sciences, Makerere University, Kampala, Uganda; ^3^ Division of Infectious Diseases and International Medicine, Department of Medicine, University of Minnesota, Minneapolis, MN, United States; ^4^ Department of Immunology and Molecular Biology, School of Biomedical Sciences, College of Health Sciences, Makerere University, Kampala, Uganda; ^5^ Division of Infectious Diseases, Department of Medicine, John Hopkins University School of Medicine, Baltimore, MD, United States; ^6^ Department of Medicine, School of Medicine, College of Health Sciences, Makerere University, Kampala, Uganda; ^7^ Mucosal and Vaccine Research Program Colorado, Department of Medicine, Division of Infectious Diseases, University of Colorado Denver, Aurora, CO, United States; ^8^ Department of Medicine and Infectious Disease, Denver Veterans Affairs Medical Center, Denver, CO, United States

**Keywords:** cryptococcal meningitis, cerebrospinal fluid (CSF), neuroimmune responses, chemokine CXCL10, cytokine IL-15, CCL11/Eotaxin, gender/biological sex survival, antifungal treatment

## Abstract

**Introduction:**

Survival among people with HIV-associated cryptococcal meningitis (CM) remains low, particularly among women, despite the currently optimal use of antifungal drugs. Cryptococcus dissemination into the central nervous system [brain, spinal cord, and cerebrospinal fluid (CSF)] elicits the local production of cytokines, chemokines, and other biomarkers. However, no consistent diagnostic or prognostic neuroimmune signature is reported to underpin the risk of death or to identify mechanisms to improve treatment and survival. We hypothesized that distinct neuroimmune signatures in the CSF would distinguish survivors from people who died on antifungal treatment and who may benefit from tailored therapy.

**Methods:**

We considered baseline clinical features, CSF cryptococcal fungal burden, and CSF neuroimmune signatures with survival at 18 weeks among 419 consenting adults by “gender” (168 women and 251 men by biological sex defined at birth).

**Results:**

Survival at 18 weeks was significantly lower among women than among men {47% vs. 59%, respectively; hazard ratio (HR) = 1.4 [95% confidence interval (CI), 1.0 to 1.9; p = 0.023]}. Unsupervised principal component analysis (PCA) demonstrated divergent neuroimmune signatures by gender, survival, and intragender-specific survival. Overall, women had lower levels of programmed death ligand 1, Interleukin (IL) (IL-11RA/IL-1F30, and IL-15 (IL-15) than men (all p < 0.028). Female survivors compared with those who died expressed significant elevations in levels of CCL11 and CXCL10 chemokines (both p = 0.001), as well as increased T helper 1, regulatory, and T helper 17 cytokines (all p < 0.041). In contrast, male survivors expressed lower levels of IL-15 and IL-8 compared with men who died (p < 0.044).

**Conclusions:**

Survivors of both genders demonstrated a significant increase in the levels of immune regulatory IL-10. In conclusion, the lower survival among women with CM was accompanied by distinct differential gender-specific neuroimmune signatures. These female and male intragender-specific survival–associated neuroimmune signatures provide potential targets for interventions to advance therapy to improve the low survival among people with HIV-associated CM.

## 1 Introduction

Co-infection with the fungus *Cryptococcus neoformans* remains an important contributor to death among people with advanced HIV/AIDS worldwide, despite the use of antifungal medications (1, 2). Mortality with HIV-associated cryptococcal meningitis (CM) varies by location based on existing HIV prevalence. In Europe and North America, with the low prevalence of people with HIV, both the incidence and mortality rates of CM are lowest (3). In contrast, in low- and middle-income countries, especially in Africa, the prevalence, incidence, and mortality with both HIV and CM are high (3–5). In Uganda, Botswana, and South Africa, the 10-week mortality rates approach 50% (3, 5–8) even in closely monitored research settings, with deaths occurring within days to weeks and, sometimes, up to months after diagnosis (2, 9, 10). These observations emphasize the importance of characterizing early immune response as a possible intervention to control fungal infection in improving survival (10).

The damage response paradigm of Casadevall and Pirofski highlights the counterbalancing contributions of pathogen vs. host response in shaping tissue injury and disease outcome (11, 12). Pathologic outcomes may result both from unrestricted pathogen growth with limited immune control or with control of the pathogen but with an exuberant immune response. This paradigm suggests that improved outcomes are achievable in the presence of therapy combining an effective pathogen-specific target drug (e.g., antifungals) with immune-based treatment to modulate immune homeostasis.

Cryptococcal dissemination into the central nervous system (CNS) tissues and cerebrospinal fluid (CSF) across the blood-brain barrier (13–15) leads to activation of resident neuroimmune cells (astrocytes, microglial cells, local macrophages, dendritic cells, and lymphocytes) and of CNS infiltrating T and B cells (16, 17). These cells produce chemoattractant proteins [e.g., CCL11/Eotaxin, CXCL10/Interferon-inducible protein 10 (IP-10) (18, 19)] and other inflammatory mediators (e.g., IL-15 and IL-8/CXCL8) (20) to induce neuroimmune activation, inflammation, and meningoencephalitis. These mediators influence fungal clearance, ensuing immunopathological processes, clinical phenotype, and outcome (21–25).

Among studies reporting cases of CM by gender (biological sex assigned at birth), the majority of CM was diagnosed among men (2, 7, 9, 16, 26–44); only four, to date, described survival by gender. Two studies done prior to optimized antiretroviral therapy (ART) initiation (delayed for 2 weeks after CM diagnosis based on the COAT Trial (9)) showed no differences in survival by gender (26, 44). In contrast, two studies done after initiation of optimized ART with antifungal therapy showed lower survival among women (7, 42). To date, no clear immunopathogenic mechanisms have been proposed to explain the divergence in survival by gender despite access to current optimal antifungal treatment and ART.

In prior studies of CM, men showed increased expression of innate chemokines and cytokines in the CSF associated with increased trafficking of innate lymphoid and myeloid cells compared with women (7). Of note, no differences were noted in soluble immune factors by survival with CM overall (7). However, these differences were not evaluated in relation to gender-specific survival. Indeed, gender-specific differences have been reported in immune responses to vaccines in both humans and animals (45, 46), with elevations in cytokine production, endocrine, and metabolic parameters in women compared with that in men. Nevertheless, in CM, the cryptococcal fungal burden, white cell counts, and protein in CSF and CD4^+^ T-cell numbers in blood were similar by gender (7). Moreover, the macrophage-mediated host immune evasion mechanism vomocytosis and cryptococcal intracellular proliferation in infected macrophages were similar by gender (47).

We evaluated differences in soluble cytokine, chemokine, and immunoregulatory responses to CM in CSF between women and men overall, by survival overall, and, in particular, the differences in these neuroimmune responses in relation to survival by gender. At the time of diagnosis, we determined levels of representative T helper 1 (Th1), Th2, Th17, T follicular helper (Tfh) cytokines, innate myeloid–regulating cytokines [interleukin-8 (IL-8), IL-13, and IL-15], and immune checkpoint markers [programmed death ligand 1 (PD-L1)] among people who survived or died during the 18-week follow-up. We identified discrete differences in patterns of neuroimmune mediators by gender and by intragender-specific survival at the site of infection in the CSF.

## 2 Materials and methods

### 2.1 Scope: participants, sites, and setting

We enrolled 419 participants from a retrospective cohort of 460 consenting adults with CM who were enrolled to receive meningitis treatment in the prior Adjunctive Sertraline for the Treatment of HIV-Associated Cryptococcal Meningitis trial (ClinicalTrials.gov: NCT01802385) and had their specimens and data in storage. The parent trial was conducted between 2015 and 2017 at the Infectious Diseases Institute (Administrative site) and at Mulago and Kiruddu National Referral Hospitals (patient catchment sites) in Kampala, Uganda (2, 48). The participants were selected on the basis of gender, available survival-specific data, and CSF specimens and clinical data in storage for analysis. All analyses were performed at baseline (cross-sectional analyses) with reference to the 18-week survival after CM diagnosis and study enrollment. The complete demographic, clinical, and CSF datasets were available from majority of participants (94%; 168 women and 251 men).

In the parent study, participants or their surrogates gave informed written and signed consent for their study participants under ethically approved study protocols. Enrolled were participants ≥18 years of age, with confirmed diagnosis of HIV-associated CM diagnosed as previously described (49, 50). Only participants whose survival status was known at study censoring and at study termination at 18 weeks of follow-up (90.7%; 380 of 419) were included in the survival sensitivity analyses.

### 2.2 Specimen preparation

CSF was drawn from lumbar punctures at diagnosis of CM prior to antifungal therapy initiation. The CSF specimens were spun to pellet out cells. The CSF supernatants were stored in a −80°C freezer prior to thawing for testing using Luminex.

### 2.3 Luminex cytokine and chemokine immunophenotyping

A representative sample of cytokines, chemokines, and checkpoint regulators was measured in CSF diluted in a 1:2 ratio based on the R&D Human XL Cytokines Discovery Premixed Kit platform as per the manufacturer’s recommendations (R&D, Minneapolis, MN). The Th1 cytokines were tumor necrosis factor–alpha (TNF-α), interferon-gamma (IFN-γ), interleukin-2 (IL-2), IL-12p70, with soluble CD40-Ligand/TNFSF5. The Th2 cytokines were IL-4 and IL-13. Tfh cytokines were IL-6 and IL-10. The Th17 cytokine included was IL-17A. Cytokines derived from innate lymphoid and myeloid cells were IL-15, IL-8, and IL-1 RA/IL-1 F3. The inflammatory-mediating chemokines primarily derived from microglial and astrocytes mediating neuroinflammation in CM were CXCL10 lymphoid cells mediating chemoattraction (51, 52) and CCL11 myeloid cells (Eosinophils) mediating chemoattraction (53, 54) and IL-8 neutrophils mediating activation and chemoattraction (55). The immune checkpoint molecules were PD-L1/B7-H1 (56) and immune regulatory cytokine IL-10 (57).

### 2.4 Statistical analysis

Data were analyzed using GraphPad Prism version 9.3.0, GraphPad Software, LLC, for Macintosh (San Diego, California, USA). The databases were compiled using Microsoft Excel. The data variability was visualized using unsupervised principal component analysis (PCA) using eigenvector covariate projection on biplots as described elsewhere (58–61). Further interrogation of the individual or independent principal component clustering and variability, factor differences, and interactions or associations of independent predictor variables by gender and by survival was performed using univariate and multivariate analyses.

Univariate analytic methods comprised pairwise comparisons using Mann–Whitney non-parametric U-test that compared arithmetic medians and unpaired parametric t-test that compared arithmetic means. In this context of non-normally distributed population variables, statistical differences were reported on the basis of the difference in the sample medians with interquartile ranges (IQRs). The univariant difference in the survival outcome or survival risk was determined using the Kaplan–Meier/log-rank test (Mantel–Cox chi-square test) or the Mann–Whitney U-test. The difference in binary outcome was determined using the Fisher’s exact chi-square test or the Mann–Whitney U-test.

In multivariate models, data were Log_2_-transformed to normalize the variable prior to the statistical interrogation using multivariate factor analysis and or survival-adjusted logistic regression least square models that measure the risk of likelihood (proportional hazard ratios). In all models, missingness was not imputed. In this context, statistical significance in both the univariate and the multivariate models was based on difference among variables in the original available dataset among participants. The statistical level of the significantly different covariates was reported at a power of 0.80, at a p-value <0.05, and at a 95% level of confidence. The rigor of our interrogations included a large sample size (N = 419), controlled comparative covariables at nearly a 1:1 ratio, and minimal missingness with approximately 94% data completeness in gender analyses and approximately 90% data completeness in the 18-week survival analyses.

## 3 Results

### 3.1 Baseline demographics by gender

**Figure 1:**
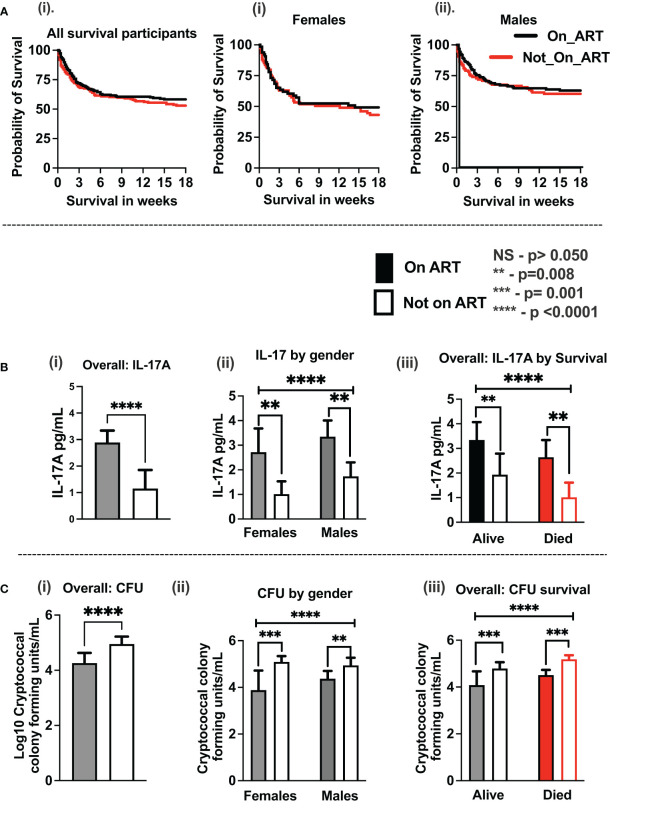
Association of baseline antiretroviral therapy on CSF immune response and host survival on antifungal treatment. **(A)** Kaplan–Meier survival by ART experience (i) and by gender (ii, iii). **(B)** IL-17A levels by ART experience (i), by gender (ii), and by host survival (iii). **(C)** CSF cryptococcal fungal burden by ART experience (i), by gender (ii), and by host survival (iii). Participants with ART status (N = 359 participants). Participants on ART (n = 188 participants) and not on ART (n = 171 participants). Females on ART (n = 65 females) and not on ART (n = 75 females). Males on ART (n = 123 males) and not on ART (n = 96 males). Asterisks (*) show statistically significant variables at p < 0.050, at 95% confidence intervals.

At enrollment, all participants with CM in the current study (419 participants) were antifungal treatment–naïve, but more than half of the participants (52.3%) were ART-experienced with the median time on ART of 1.6 months (IQR, 0–22 months). However, no statistical difference in survival was observed between ART-naïve and ART-experienced participants overall and by gender (**Figure 1A**). The cytokines levels showed elevation with ART with IL-17A being statistically significant overall, by gender and by survival (**Figure 1B**). The cryptococcal CSF fungal burden was significantly lower with ART overall, by gender and by survival (**Figure 1C**). Survival in the parent cohort was at 50.9% (2), and survival did not differ statistically between either sertraline randomized participants or those on standard therapy alone (2).

**Figure 2:**
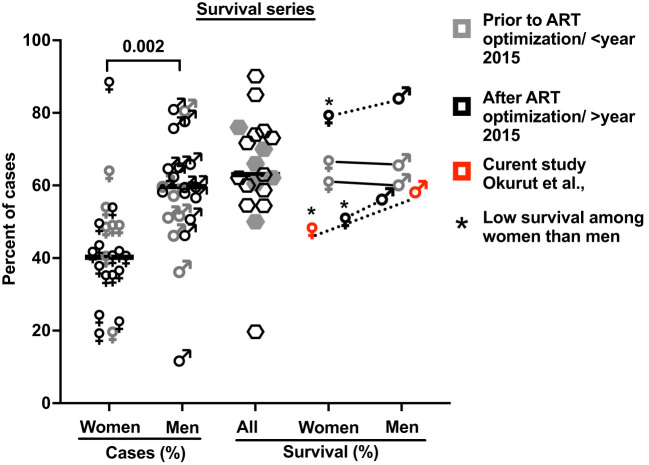
The proportion of cases with HIV-associated CM by gender and of related survival by gender in 21 published case series. These studies include 38,485 cases with 5,834 reported deaths, which accounted for a 15.2% case fatality rate (see **Supplementary Table 1** for details and references (7, 9, 16, 26, 28–44)). Bars show median values. Five studies report survival by gender (four references and this report; **Supplementary Table 1**). * Shows published data with significantly low survival among women treated for cryptococcal meningitis compared to men.

Consistent with published reports (**Figure 2**), among the 419 participants studied with HIV-associated CM, the majority were men, who were older and heavier than women (**Table 1**). Neurologic abnormalities predominated among clinical signs and symptoms, with almost all describing headache for 1 to 4 weeks, a third reporting changes in mental status, and a half with abnormal Glasgow Coma Score (GCS < 15), with each variable reported at comparable frequencies in men and women.

**Table 1:** Baseline demographics of people with HIV-associated cryptococcal meningitis by gender.

Variables	N	Women Median (IQR) or N (%)	N	Men Median (IQR) or N (%)	P-value
**Participants, N = 419, n (%)**		168 (40.1)		251 (59.9)	
Demographics					
Age, years	167	32 (28–38)	246	35 (30–40)	0.004
Weight, kg	155	50 (47–60)	238	54 (50–60)	0.007
On ART, n (%)	167	73 (48.7)		125 (54.3)	
Duration of ART, days	77	167 (64–1,109)	135	231 (29–1,095)	
Signs and symptoms					
Headache at presentation, n (%)	168	161 (95.8)	251	240 (95.6)	
Duration of headache, days	161	14 (7–21)	240	14 (7–30)	
Cachexia, n (%)	167	72 (43.1)	246	112 (45.5)	
Photophobia, n (%)	166	57 (34.1)	145	66 (26.8)	
Seizures present, n (%)	168	22 (13.1)	246	42 (17.1)	
Altered mental status, n (%)	167	56 (33.5)	246	96 (39)	
Glasgow Coma Scale <15, n (%)	166	80 (47.9)	246	115 (46.7)	
Systolic blood pressure, mmHg	166	120.0 (105.8–131.3)	242	123.0 (112.8–138.3)	0.007
Diastolic blood pressure, mmHg	166	79.5 (69.0–90.0)	242	80.0 (70.0–93.0)	
Blood clinical analysis					
Glucose, mmol/L	75	5.8 (4.9–6.8)	111	5.7 (4.9–6.4)	
White blood cells, ×10^9^/L	156	3.5 (2.7–4.9)	226	3.5 (2.5–5.1)	
CD4 T-cell counts/µL	162	23 (8–58)	234	15 (5–39)	0.004
CD8 T-cell counts/µL	160	334 (168–632)	230	283 (172–481)	
Hemoglobin, g/dL	156	10.8 (9.1–12)	226	12.4 (10.4–13.7)	<0.001
Platelets, ×10^9^/L	156	216 (144–270)	225	179 (128–245)	0.021
CSF Clinical analysis					
Glucose, mmol/L	55	3.3 (2.1–4.4)	78	4.6 (2.3–6)	0.033
Protein mg/dL	140	40 (23–96)	199	58 (25–113)	
White blood cells/µL	160	<5 (<5–34)	241	<5 (<5–45)	
Opening pressure, mmH_2_O	143	270 (180–424)	212	298 (220–427.5)	
Cryptococcal culture, Log_10_ CFU/mL	152	4.9 (3.3–5.6)	223	4.7 (3.7–5.6)	

Statistics: Mann–Whitney U-test, chi-square test. Not statistically significant variables had p-value ≥0.05 at a 95% confidence interval. CFU, colony-forming units; CSF, cerebrospinal fluid; ART, antiretroviral therapy. Normal CSF proteins in adults, 15–60 mg/dL; normal CSF glucose, 2.5–4.4 mmol/L.

As anticipated, CD4^+^ T-cell numbers in circulation were low but were marginally higher in women than that in men, as were platelet counts and hemoglobin levels (**Table 1**). CSF protein was not consistently elevated, and white blood cells (WBC) counts were low, despite a high burden of yeast. Each result was generally comparable by gender, except CSF glucose that tended to be lower among women than that among men (**Table 1**).

### 3.2 The 18-week survival on antifungal treatment

**Figure 3:**
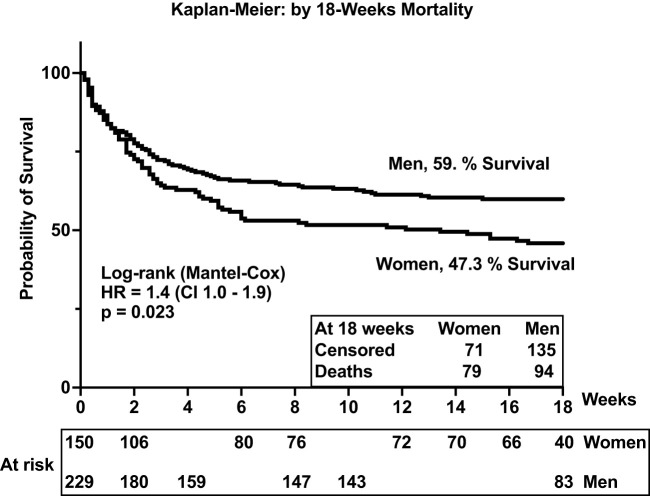
Survival among people with HIV-associated cryptococcal meningitis. The p-value <0.05 is statistically different.

In the parent trial for this analysis, men and women from Uganda and South Africa were randomized 1:1 to receive sertraline (an antidepressant with putative antifungal activity) or standard treatment but showed no differences in survival between treatment groups at 18 weeks (2). An initial analysis by gender of an expanded data set, from which this current report is derived, showed lower 10-week survival among 400 women vs. 577 men (at 50% female survival vs. 57% male survival, respectively), although at 10 weeks unadjusted [hazard ratio (HR) was only borderline, HR = 1.20; 95% confidence interval (CI), 1.00–1.45; p ≤ 0.050] (7). These differences were greater yet, in this current subset, extended to 18 weeks of observation. Survival among women was 47% (71 of 150 women) vs. 59% among men (136 of 230 men) [Mantel–Cox proportional HR = 1.4 (95% CI, 1.0–1.9); p = 0.023) (**Figure 2**). Of note, survival was similar among men and women in two earlier studies reported prior to ART optimization (before 2015) but lower among women in two studies reported after ART optimization (after 2015), which include the current report (**Figures 2**, **3**).

Survival was similar by gender in the first 2 weeks of antifungal therapy (**Figure 2**). However, survival diverged thereafter, remaining consistently lower among women throughout the 18 weeks of observation. As noted, baseline demographics, signs and symptoms, blood and CSF analytes, and cryptococcal fungal burden were relatively similar among participants by gender and by survival (**Table 1**). Thus, we considered whether the concentrations and the composition of neuroimmune induced factors at the site of severe cryptococcal disease in the CSF could underlie subsequent differences in survival by gender over time of observation.

### 3.3 Significant differences in neuroimmune signatures in cerebrospinal fluid by survival, gender, and intragender-specific survival

**Figure 4:**
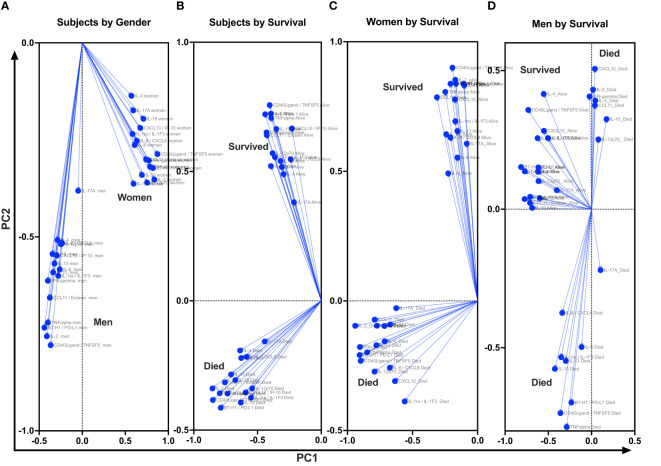
Projection of cytokine responses on eigenvector correlation covariates on PC1 and PC2 (axes) using unsupervised principal component analysis. Baseline cerebrospinal fluid (CSF) immune signature among participants who were diagnosed with HIV-associated CM showed distinct clusters by gender and by survival. PC, principal component. Dots show individual cytokines produced among participants projected on eigenvector correlation covariates. Variables near the center (0; zero) are uncorrelated to the model, and variables further away from the center (0; zero) are strongly correlated to the model. Variables among all cytokine projected on the variety of CSF cytokine patterns among participants by host survival (**Figure 3A**). The CSF secreted cytokines clustering by gender (**Figure 3B**). Cytokine clustering by female survival (**Figure 3C**). Cytokine clustering by men’s survival (**Figure 3D**).

At baseline, we performed unsupervised PCA as a primary approach to visualize the data variability and to explore potential unbiased differences in data clustering by gender, survival, and gender-specific survival (**Figure 4**). The PCA identified individual clusters that offered opportunities to structure downstream data analyses of the indicated model outcomes. The members in the cluster aggregated on the basis of common attributes of the datasets showing high variability and distinct distribution of cytokines and chemokines between women and men (**Figure 4A**), between survivors and participants who died (**Figure 3B**), and within gender survival, among women (**Figure 4C**) and among men (**Figure 4D**). Eigenvector projections on principal components 1 and 2 (PC1 and PC2, respectively) indicated a high probability of neuroimmune variables predicting association with gender and survival outcome (**Figures 4A–D**). High eigenvalues >5 indicate the high capability of the selected covariables in predicting model-associated outcomes (**Table 2**). For each of the comparative groups, almost all showed such high eigenvalues and, thereby, the separation between determinants in each group. These distinct patterns for PC1 and PC2 were closely correlated among participants by gender, subject by survival, and women by survival but less so for male survival in which three clusters were identified (correlation data not shown). Because of the notable data clustering observed in these groups, we next determined the specific neuroimmune factors contributing to the observed patterns using supervised univariate and multivariate data interrogation approaches.

**Table 2:** Adjacent Table to **Figure 4**.

Group Analyzed	Participants by gender		Participants by Survival		Women by Survival		Men by Survival	
**PC summary**	PC1	PC2	PC1	PC2	PC1	PC2	PC1	PC2
**Eigenvalue**	9.8	7.3	9.5	7.6	10.1	9.5	7.6	4.7
**Proportion of variance (%)**	30.6	22.7	29.7	23.9	31.7	29.7	23.9	14.6
**Cumulative proportion of variance (%)**	30.6	53.3	29.7	53.6	31.7	61.3	23.9	38.5

PC1 shows the highest variance of loading on a single vector. PC2 shows the cumulative variance of loading that is orthogonal to PC1 with a center 0. Yet negative variables show the presence of hidden (latent) variables that can be only determined through inference using mathematical modeling or through direct measurement alongside observed variables (those with positive variance on PCA). The greater the eigenvalues than one (1), the greater the predictability power of the variable in determining the hidden or latent variance to the outcome.

### 3.4 Innate neuroinflammatory cytokines in the cerebrospinal fluid differ by gender

The CSF of women with CM at baseline had significantly lower levels of selected innate cytokines than that of men, particularly IL-1RA and IL-15, TNF-α and immune checkpoint, and PD-L1 (all p < 0.050) (**Table 3**). The remainder of the cytokines and chemokines interrogated did not differ significantly by gender but tended to be lower still among women than that among men (**Table 3**). We next considered whether such gender-specific differences were associated with the differences in survival by gender.

**Table 3:** Differences in baseline immune signature in cerebrospinal fluid exudate by gender among patients with HIV-associated cryptococcal meningitis.

Variables	Women, Median (IQR)	Men, Median (IQR)	P-value
Participants, N = 419	168 (40.1)	251 (59.9)	
Immune checkpoint inhibitor			
PD-L1/B7-H1	90.0 (41.0–170.6)	112.5 (53.4–205.9)	0.028
T helper 1 cytokines			
TNF-α	37.4 (11.8–86.3)	49.2 (20.3–109.4)	0.048
IL-2	5.0 (2.1–8.8)	5.1 (2.5–9.0)	
IFN-γ	3.8 (0.4–10.3)	5.2 (0.4–12.9)	
CD40 Ligand/TNFSF5	370.2 (73.1–636.4)	411.1 (188.2–715)	
CCL11/Eotaxin	15.0 (6.2–20.7)	14.5 (7.4–20.2)	
IL-12p70	5.2 (1.4–10.0)	6.0 (1.7–10.4)	
CXCL10/IP-10	2,407.0 (1,038.0–3,037.0)	2394.0 (1,161.0–3,017.0)	
T helper 2 cytokine			
IL-4	1.2 (0.4–2.2)	1.3 (0.5–2.3)	
IL-13	22.3 (10.9–35.4)	21.9 (10.6–35.0)	
T helper 17 cytokine			
IL-17A	1.6 (0.2–4.1)	2.3 (0.2–5.4)	
T follicular helper cytokines			
IL-10	213.2 (144.7–325.2)	242.8 (161.6–348.3)	
Innate myeloid cytokines			
IL-1RA/IL-1F3	4041.0 (1,139.0–10,005.0)	6142.0 (2,582.0–10,067.0)	0.001
IL-15	2.8 (1.8–4.6)	3.6 (2.5–5.2)	0.003
IL-8/CXCL8	346.5 (148.0–1042.0)	380.4 (135.4–1,118.0)	
IL-6	142.4 (44.4–721.3)	209.3 (48.6–1,344.0)	

Cerebrospinal fluid cytokine differentiation with gender: Cytokine and chemokine levels were measured in picogram per milliliter (pg/mL). Statistics: Mann–Whitney U-test. The statistically similar variable had a p-value ≥0.05 at a 95% confidence interval.

### 3.5 Divergent baseline neuroimmune cytokine signatures predict intragender survival

#### 3.5.1 Female gender–specific survival attributes

A number of relevant factors differed between women who survived or died during the 18-week observation. The circulating CD4^+^ T-cell numbers were generally very low, but median CD4^+^ T cells were 31 cells/µL vs. 14 cells/µL among women who survived vs. those who died (p = 0.009) but did not differ by survival in men (data not shown). Using several alternative models and after adjusting for cytokines (**Table 4**, model 1), CXCL10 and CCL11 consistently predicted female survival but not in men. These soluble immune factors were consistently higher in magnitude among female survivors compared with those who died (**Table 5**). The level of CXCL10 was significantly higher among women who survived than those who died (p = 0.013) (**Table 5**). By survival, CXCL10 levels in women who survived correlated with the number of CSF white cell counts (r = 0.292; 95% CI, 0.067–0.488; p = 0.010) but not in men (**Supplementary Figure 1**). The fungal burden [colony-forming units (CFUs)] did not correlate with CXCL10 levels by either gender or gender-specific survival (data not shown).

**Table 4:** Baseline-independent factors predicting intragender 18-week survival among patients with HIV-associated cryptococcal meningitis on antifungal therapy.

Variables Cytokines, Log_2_ (Pg/mL)	Women Odds ratio estimate (95% CI for profile likelihood)	P-value	Men Odds ratio estimate (95% CI for profile likelihood)	P-value
**Survival at 18 weeks, N (%)**	71 of 150 (47.3)		136 of 230 (59.1)	0.024
**N (range)/model**	Survived (n = 63 to n = 70) Died (n = 60 to n = 71)		Survived (n = 118 to n = 131) Died (n = 78 to n = 91)	
Model 1: survival adjusted for cytokine				
**CCL11/Eotaxin**	0.42 (0.20–0.80)	0.014	1.65 (1.00–2.76)	
**CXCL10/-IP-10**	0.48 (0.27–0.79)	0.006	1.03 (0.76–1.40)	
**IL-8/CXCL8**	0.93 (0.65–1.31)		1.38 (1.03–1.88)	0.034
**IL-15**	0.96 (0.57–1.62)		1.88 (1.20–3.04)	0.007
Model 2: survival adjusted for age, weight, systolic blood pressure and cytokines				
**CCL11/Eotaxin**	0.35 (0.16–0.69)	0.005	1.55 (0.94–2.60)	
**CXCL10/IP-10**	0.55 (0.30–0.96)	0.042	1.00 (0.73–1.37)	
**IL-10**	2.26 (1.00–5.11)	0.044	0.65 (0.32–1.27)	
**IL-15**	0.86 (0.48-1.50)		1.63 (1.03–2.64)	0.040
Model 3: survival adjusted for hemoglobin, platelets count, CD4 counts, and cytokines				
**CD4 T-cell counts/µL**	0.99 (0.98–1.00)	0.039	1.00 (0.99–1.01)	
**IL-2**	1.94 (1.13–3.78)	0.025	0.59 (0.30–1.06)	
**IL-10**	2.33 (1.04–5.39)	0.038	0.73 (0.34–1.51)	
**CCL11/Eotaxin**	0.29 (0.12–0.63)	0.004	1.044 (0.970–1.129)	
**CXCL10/IP-10**	0.45 (0.25–0.76)	0.004	0.98 (0.69–1.38)	
**IL-8/CXCL8**	0.92 (0.64–1.32)		1.69 (1.17–2.58)	0.009
**IL-15**	0.81 (0.43–1.49)		1.79 (1.05–3.20)	0.041
**Hemoglobin, g/dL**	0.82 (0.66–1.02)		0.82 (0.70–0.94)	0.006
Model 4: survival adjusted for sodium, potassium, and cytokines				
**CCL11/Eotaxin**	0.32 (0.12–0.70)	0.009	1.74 (1.01–3.05)	0.048
**CXCL10/IP-10**	0.52 (0.27–0.92)	0.033	1.10 (0.79–1.55)	
**IL-8/CXCL8**	0.87 (0.59–1.28)		1.41 (1.02–2.00)	0.044
**IL-15**	0.89 (0.48–1.66)		2.36 (1.43–4.12)	0.001
**Sodium, mmol/L**	0.96 (0.88–1.04)		0.87 (0.93–0.98)	0.011
**Potassium, mmol/L**	0.49 (0.24–0.95)	0.044	0.99 (0.62–1.59)	

Multivariate adjusted models of gender-specific associated factors (univariate gender-specific survival–associated factors in **Table 5**) were interrogated with statistically different gender-associated demographic variables (**Table 1**). Odds ratios represent the independent likelihood of survival based on levels of the indicated factor with an adjusted multivariate logistic regression model. Statistic: intragender (male or female) multiple logistic regression. Not statistically significant variables had adjusted p-value ≥0.05 at a 95% confidence interval.

**Table 5:** Univariate difference in baseline levels of CSF factors associated with gender survival among patients with HIV-associated CM.

Cytokine, Pg/mL	Women Alive Median (IQR)	Women Died Median (IQR)	P-value	Men Alive Median (IQR)	Men Died Median (IQR)	P-value
**N**	71	79		136	94	
T helper 1 cytokines						
**IL-2**	6.0 (4.4–10.1)	4.3 (2.0–7.7)		4.5 (2.3–8.6)	5.2 (2.8–10.3)	
**IFN-γ**	5.2 (0.4–14.0)	2.1 (0.3–8.4)		3.8 (0.4-10.9)	6.0 (0.4–16.2)	
**IL-12p70**	7.6 (2.6–11.2)	3.6 (0.8–8.4)	0.018	6.0 (1.6–10.2)	6.0 (2.0–10.8)	
**TNF-α**	54.1 (15.8–108.5)	27.9 (8.3–69.4)	0.041	61.1 (27.4–104.0)	38.1 (15.4–115.5)	
**CD40-L/TNFSF5**	428.1 (115.7–732.7)	290.4 (65.2–534.7)	0.049	433.7 (171.2–710.9)	395.2 (188.2–797.4)	
**CCL11/Eotaxin**	17.0 (11.4–21.6)	12.4 (3.3–18.6)	0.001	14.5 (7.2–20.1)	14.6 (9.2–20.5)	
**CXCL10/-IP-10**	2,613.0 (2,071.0–3,330.0)	1,590.0 (497.1–2,702.0)	0.001	2,515.0 (1,432.0–3,016.0)	2,369.0 (974.1–3,068.0)	
T helper 2 cytokines						
**IL-4**	1.3 (0.6–2.6)	1.1 (0.3–2.1)		1.3 (0.5–2.5)	1.6 (0.7–2.3)	
**IL-13**	28.7 (14.0–38.1)	20.1 (5.9–31.6)	0.041	27.6 (18.4–37.6)	20.6 (4.2–32.5)	0.001
T helper 17 cytokines						
**IL-17A**	2.8 (0.9–6.0)	0.9 (0.2–3.0)	<0.001	2.8 (0.3–5.9)	2.2 (0.2–5.1)	
T follicular helper or immune regulatory cytokine						
**IL-10**	241.2 (175.7–384.5)	184.1 (175.7–278.1)	0.009	267.0 (209.7–374.7)	212.4 (134.5–338.8)	0.002
Innate-like cytokine						
**IL-1RA/IL-1F3**	3830.0 (12,070.0–10,005.0)	3983.0 (843.1–10,011.0)		5361.0 (2,329.0–10,032.0)	6201.0 (2,378.0–10,078.0)	
**IL-6**	155.7 (36.3–962.1)	123.4 (40.6–514.5)		202.4 (48.3–1,267.0)	189.0 (41.6–1,305.0)	
**IL-8 (CXCL8)**	460.0 (147.8–1,042.0)	303.5 (141.6–916.4)		329.1 (162.9–938.3)	384.9 (112.4–1,153.0)	
**IL-15**	2.8 (1.8–5.3)	2.7 (1.7–4.0)		3.2 (2.3–4.6)	4.0 (2.9–5.9)	0.002
Immune checkpoint factor						
**PD-L1**	107.3 (44.2–174.9)	72.2 (36.0–158.5)		126.9 (67.1–192.0)	101.1 (50.5–216.6)	

Cerebrospinal fluid cytokine differentiation with gender-specific survival: Statistics: Mann–Whitney U-test, not statistically significant variables had a p-value ≥0.050 at a 95% confidence interval.

The level of CCL11 (a chemoattractant produced by activated astrocytes, lymphocytes, and macrophages) was significantly higher in female survivors than in those who died but not among men (**Table 5**). After adjusting for other cytokines, higher CCL11 expression still predicted female but not male survival (**Table 4**, model 1). T-cell–related factors (e.g., IL-12p70, IL-17A, and IFN-γ) in the univariate models were also consistently increased in women who survived compared with those who died but not in men (**Table 5**). The levels of other cytokines and the neutrophils chemokine IL-8 showed no difference among women by survival (**Table 5**). Only IL-10 level was increased in both genders among those who survived in univariable analysis (**Table 5**). However, after adjusting for other factors, the levels of IL-10 expression were correlated with only female survival, but not with male survival (**Table 4**, models 2 and 3), as was IL-2 (**Table 4**, model 3).

In summary, although immune parameters were lower among all women vs. men, the women who survived showed consistently elevated levels of both myeloid-derived chemokines (CCL11), lymphoid-derived chemokine (CXCL10), T-cell–derived cytokines, and the regulatory molecule PD-L1, the differences that were mostly distinct to survival in women but not in men (**Supplementary Figure 1**).

#### 3.5.2 Male gender–specific survival attributes

Men who survived had significantly higher hemoglobin compared with those who died, even after adjusting for the platelet counts and CD4^+^ T-cell counts (**Table 4**, model 3). Men, but not women, who survived had significantly lower IL-15 than those who died (**Table 5**), even after adjusting for other cytokines (**Table 4**, all models). The IL-15 levels were independent of fungal burden (CFUs) and CSF white cell counts (data not shown). After adjusting for other factors, lower levels of the neutrophil chemoattractant IL-8 also consistently predicted male survival but not among women (**Table 4**, models 1, 3–4). Unlike in men, low levels of neither IL-15 nor IL-8 were associated with survival in women. As noted above, elevated levels of regulatory IL-10 were associated with survival in both genders but did not correlate with male survival (**Supplementary Figure 1**).

## 4 Discussion

In a large cohort of adults with CM in Uganda on optimized ART anti-fungal therapy, survival among women was significantly lower compared with that among men. Evaluating gender-specific mortality is uncommon in this context (only five including the current of 21 studies; **Supplementary Table 1**). Unlike in the two previous studies that showed no difference in mortality by gender reported (26, 44), the two studies showing such a difference (7, 42) were performed in people treated with optimally timed (delayed) initiation of ART that lowers mortality and with optimized antifungal therapy, which helps control fungal burden. Combining antifungal drugs with immunomodulatory interventions as new therapies could limit the fungal and immune assault on the CNS. Illustrated by high mortality among CM-infected subjects with more prominent brain lesions from magnetic resonance imaging and at autopsy who could benefit from combining antifungal drugs with immunomodulatory interventions (62–65). As proposed by Casadevall and Pirofski (11, 12), with appreciable control of fungal burden, immune responses may be a more prominent determinant of clinical outcomes.

We have identified distinct immunologic differences in CSF by gender and gender-specific survival. The signatures associated with survival in women are distinct from those in men. These differences were independent of baseline clinical features and cryptococcal fungal burden (CFUs). Most consistent in both the univariate and in multivariate gender-associated survival predictive models were the lower levels of CSF CXCL10/IP-10 and CCL11/Eotaxin that distinguished women who died from survivors but not among men. In contrast, higher levels of IL-15 and IL-8 differentiated men who died from those who survived but not among women. Several T-cell cytokines (IFN-γ, TNF-α, IL-13, and IL-17A) similarly exhibited diminished levels of expression in women who died matched survivors but not in men. Lower levels of immune regulatory IL-10 expression were linked with an increased frequency of death in both genders. Thus, the biological significance of gender-specific CSF immune signatures identified among participants with CM may underlie important immune-based mechanisms as a guide to improving antifungal treatment and survival predominantly in women.

Consistent with our univariate model observations, prior studies showed that improved survival at 2–10 weeks was associated with elevation in the levels of soluble CSF cytokines Th1 IL-2, IFN-γ, TNF-α, Th2 IL-4, and Th17 IL-17A, as well as innate IL-6 (66, 67). Soluble T-lymphokine levels in CSF are proposed to derive from CXCL10-mediated (68) and CCL11-mediated (69) stimulation of CNS-resident immune cells. Moreover, the use of cryptococcal antigens [such as Glucuroxylomannan (GXM)] to stimulate peripheral immunocytes demonstrated that patients with improved 10-week survival had increased expression of these cytokines among polyfunctional differentiated CD4 T cells (67). Other chemokines reported to predict improved survival included the elevated levels of monocytes chemotactic protein-1 and macrophages inflammatory protein-1 (66). Despite the demonstrated correlations of survival in the peripheral circulation (70, 71), the importance of characterizing immune responses in the CNS, at the foci of infection, cannot be underestimated, because CNS responses in larger studies do not often correlate with those in the peripheral circulation (70, 71). The disparity between peripheral and CNS observed responses is a characteristic indicator of local mechanisms that influences responses. Compartment-specific responses have been noted previously (16, 17) and highlight the relevance of characterizing the response in CSF. Thus, the current study is unique in characterizing the distinctive neuroimmune signatures defining survival by gender in CNS, at the foci of disease.

In theory, the microbial invasion of the CNS activates resident immune cells to produce neuroimmune cellular activating cytokines, chemoattractant chemokines, and surrounding tissue basal cellular immune mediators (prostaglandins, leukotrienes, etc.). These diverse effector cells include resident microglial, astrocytes, oligodendrocytes, CNS surveillance phagocytes (monocytes, macrophages, and neutrophils), dendritic cells, adaptive T and B cells, innate [natural killer (NK) cells], and basal barrier epithelial cells (72). Other cells may be drawn by extravasation and diapedesis through the protective vascular barriers to infiltrate the CNS in response to infection and/or to ensuing meningoencephalitis (16, 17). The consistent use of antifungal therapy in this study helps to control fungal replication and the burden of fungi (2, 9, 26, 73). With relatively effective antifungal therapy, modeling immune factors highlights the role of the immune system in determining immune-mediated damage vs. protection in the CSF and, ultimately, disease outcome (67, 74, 75).

Although cryptococcal fungal burden and other clinical parameters in blood or in CSF did not differ by gender or gender-associated survival, increased production of IL-1RA, IL-15, and PD-L1 in the CSF may have contributed to the mortality of men but not women. Lower male, but not female, survival was associated with high levels of myeloid cytokines, including the pleiotropic IL-15 and neutrophil-activating factor IL-8. In the literature, IL-8 expression derived from microglia in CNS in response to neuroinflammation (76, 77) from monocytes and autocrine IL-8 production by neutrophils enhances immune activation and cellular proliferation (20). Elevated intrathecal IL-15 and IL-8 in the CSF can enhance neutrophil-induced NETosis (neutrophils traps), which may propagate thrombotic vasculitis, cryptococcomas, and fungal occlusions in subarachnoid ventricular and arterial spaces, thereby reducing survival. Together, IL-15 and IL-8 elevation can increase local inflammation in the brain, leading to vasculitis in subarachnoid spaces; interfering with blood supply; promoting ischemia, necrosis, and infarction of the brain tissue; and potentially leading to increased risk of death. Thus, the relatively high levels of IL-15 expression among men who died and the inverse relationship of IL-15 levels with those of immune regulatory IL-10 and immune checkpoint PD-L1 among men who died highlight this dynamic functional interrelationship. Lowering levels of IL-15 may limit the mitotic division of fungal-infected macrophages that may attenuate the propagation of endogenous pathogens in quiescent cells (20), leading to lower fungal burden and improved survival.

Among neutrophil-activating factors (e.g., IL-8 and IL-15) may promote neutrophil-induced tissue injury through self-directed host and pathogen-mediated mechanisms (78, 79). Neutrophil activation is associated with the propagation of tissue necrosis, hypoxia, and nutritional supply impairment, especially among people with sepsis and multiple organ failure, which are often complicated by brain injury, e.g., stroke (78, 79). In this context, activation of neutrophil recruiting cytokines and chemokines at the foci of infection in the CNS is detrimental to the host (76, 78). Released toxic granules are destructive to connective tissues, leading to vasculitis with tissue necrosis, infarction, hypoxia, and, potentially, death. Indeed, in CM, neutrophilia in the circulation was associated with poor survival outcome (80), brain hypoxia (15, 81, 82), subarachnoid blood vessel occlusion (83), brain tissue necrosis, and cerebellar infarction (64). Subsequently, survivors of CM suffered long-term CNS abnormalities associated with impaired faculty observed in sequelae among people without HIV infection (62, 84). The persistent CNS abnormalities in sequelae noted in humans with fungal infections were similar to those observed among mouse models of Alzheimer’s disease (85). In other fatal CNS infections, such as tuberculosis and bacterial meningitis, neutrophils account for the majority of cells in the CSF (86, 87). However, neither we nor others have characterized in detail the neutrophil myeloid lineages and role among CSF white cells in the setting of CM-associated survival.

Among key differences in female intragender survival with CM, survivors tended to shed relatively higher amounts of neuroimmune-mediating soluble cytokine and chemokine responses across the panel. The higher levels of Th1 IL-2, IFN-γ, and TNF-α, as well as IL-12p70, Th17 IL-17A, and immune regulatory IL-10 may influence survival in women by distinct CXCL10 and CCL11 chemokine-stimulated mechanisms. In contrast to women who died, women with higher levels of the myeloid chemokine CCL11 and the lymphoid chemoattractant CXCL10 were more likely to survive, but not men. CXCL10 produced by astrocytes and microglia cells or by CNS-resident monocytes, macrophages, and dendritic cells stimulate Th1 cytotoxic CD8 T cells, immune modulatory CD4 T cells, regulatory T cells, NK and regulatory B cells, and regulatory T cells via its CXCR3 receptor–mediated stimulation (88). The cytotoxic response to infected macrophages is potentially enhanced in the presence of elevated chemokines in the CNS, because the infected cells respond to chemoattractant proteins at the site of infection (89, 90), whereas immune regulatory cells can modulate inflammation and cellular activation to stimulate tissue recovery (91). Thus, independent of the baseline CSF cryptococcal burden (CFUs) and the baseline clinical features, the CCL11 and CXCL10 balance is critical in immune regulation of the Th1/Th2/17 balance, modulation of CNS cellular influx that is important in better survival of host with HIV-associated CM.

IL-12 is observed as an important factor in the immune control of *Cryptococcus* meningitis as it is observed to influence mechanisms that inhibited cryptococcal fungal replication among *Cryptococcus*-infected macrophages *in vitro* (92). The timing of immunotherapy is likely important. In mouse models, early preemptive use of IL-12 as an adjuvant promoted survival of mice in early use after cryptococcal fungal (92), whereas the late treatment of mice with IL-12 after establishment of cryptococcal infection resulted in more mice dying from fungal infection attributable deaths (92). In the current study, IL-12p70 was elevated among female, but not in male, survivors. In the CNS, IL-12, produced prominently by dendritic cells, regulates T cells, including differentiation of the Th1 T cells and NK-cell function (93). With the prominence of T cells and B cells in the CSF with CM (16, 17), increased IL-12p70 expressed in the CSF could enhance fungal-specific immunocyte activation to control infection and improve host survival.

The Th17 cytokine, IL17A, modulated through RORγt regulatory pathway, works in tandem with Th1-modulated cytokines to regulate neuroimmune responses to CNS infections (94). In particular, IL-17A activates Th17 immunocytes in response to tissue-resident infection. In a cryptococcal model infection, IL-17 promoted the development of a majority of giant cryptococcal cells that have limited ability to cross the blood-brain barrier, leading to localized cryptococcal fungal infection with limited spread to the brain parenchyma (65, 95). Among women in our study, IL-17A was upregulated among those with better survival outcomes. Other Th1 cytokines upregulated among female survivors included IFN-γ and TNF-α and their immune-activating factor, CD40 ligand. These factors facilitate the formation of reactive phagosomes and the activation of reactive oxygen and nitrogen species that mediate endogenous killing of *Cryptococci* ingested by macrophages (65, 95). Collectively, these complementary immune-activating factors were each increased in association with increased survival among women. In aggregate, these novel findings, first reported herein in the setting of CM gender-specific survival, direct attention to how these results may be harnessed for targeted gender-specific immune therapy to optimize survival, which appears to operate independent of the fungal burden, clinical presentation, and current antifungal therapy.

## 5 Conclusion

Survival from HIV-associated CM remains significantly lower among women than that among men. We found novel female and male biological sex-distinctive survival associated CSF neuroimmune chemokine and cytokine biomarker signature at the time of HIV-associated CM clinical presentation (at diagnosis) that were independent of the baseline fungal burden and clinical features. Increased concentration of CCL11, CXCL10, and T-cell subset–associated cytokines (mediating the Th1 and Th17 pathways) was found to be uniquely associated with female intragender survival but not in men. In addition, low levels of the neutrophil-activating chemokine IL-8 and pleiotropic cytokine IL-15 were distinctively associated with male intragender survival but not in women. Increased expression of IL-10 and PD-L1 immune regulatory molecules were associated with improved survival in both genders. The increases in CCL11, CXCL10, IL-17A, and IL-12p70 neuroimmune cytokine signatures in CSF among women who survived and these markers’ positive correlation with IL-10 and PD-L1 highlight the interdependence of these biomarkers responses in shaping immune homeostasis and host survival. These novel neuroimmune biomarkers’ observations reveal the promising signals to be considered in attempts to model more robust biological sex-specific responses to improve HIV-associated CM treatment, clinical outcomes, and host survival.

## 6 Limitations

One limitation is that the cross-sectional, exploratory study design generated data may not explain marker and clinical variations after the baseline and prior to death outcome or study censor among survivors. The missed observations might be key to explaining the factors that underlie the observed gender-survival subjective mechanisms. The differential of myeloid vs. lymphoid white cells in the CSF compartment was largely unavailable, nor was the identification of the specific local cells producing the immune mediators. The limitations of our interpretations of the baseline cross-sectional findings can be enhanced with longitudinal data to appropriately capture and model changes in the immune profile from diagnosis to eventual survival or death.

## Author’s note

An abstract CROI 2023 poster 498 from this work was presented at the Conference on Retroviruses and Opportunistic Infections at Seattle Convention Center, Seattle, Washington, 19-22 February 2023 and was published online on CROI website 96 and part of the doctoral thesis 2023 at the Makerere University 97.

## Data availability statement

The original contributions presented in the study are included in the article/**Supplementary Materials**, further inquiries can be directed to the corresponding author.

## Ethics statement

The studies involving humans were approved by Makerere University, School of Biomedical Sciences, College of Health Sciences, Higher Degrees Research, and Ethics Committee. The studies were conducted in accordance with the local legislation and institutional requirements. The human samples used in this study were acquired from primarily isolated as part of your previous study for which ethical approval was obtained. Written informed consent for participation was provided from the participants or the participants’ legal guardians/next of kin in accordance with the national legislation and institutional requirements.

## Author contributions

SO: Conceptualization, Data curation, Formal Analysis, Funding acquisition, Investigation, Methodology, Project administration, Resources, Software, Supervision, Validation, Visualization, Writing – original draft, Writing – review & editing. DB: Conceptualization, Data curation, Formal Analysis, Funding acquisition, Investigation, Methodology, Project administration, Resources, Software, Supervision, Validation, Visualization, Writing – original draft, Writing – review & editing. EO: Conceptualization, Data curation, Investigation, Methodology, Resources, Validation, Visualization, Writing – review & editing. JR: Conceptualization, Data curation, Formal Analysis, Funding acquisition, Investigation, Methodology, Project administration, Resources, Software, Supervision, Validation, Visualization, Writing – original draft, Writing – review & editing. HK: Conceptualization, Investigation, Methodology, Project administration, Resources, Supervision, Validation, Visualization, Writing – review & editing. BB: Conceptualization, Formal Analysis, Investigation, Methodology, Project administration, Resources, Supervision, Validation, Visualization, Writing – original draft, Writing – review & editing. FB: Conceptualization, Investigation, Methodology, Project administration, Resources, Supervision, Validation, Visualization, Writing – review & editing. JO: Conceptualization, Data curation, Formal Analysis, Investigation, Methodology, Project administration, Resources, Supervision, Validation, Visualization, Writing – original draft, Writing – review & editing. YM: Conceptualization, Data curation, Formal Analysis, Funding acquisition, Investigation, Methodology, Project administration, Resources, Software, Supervision, Validation, Visualization, Writing – original draft, Writing – review & editing. DM: Conceptualization, Data curation, Formal Analysis, Funding acquisition, Investigation, Methodology, Project administration, Resources, Software, Supervision, Validation, Visualization, Writing – original draft, Writing – review & editing. EJ: Conceptualization, Data curation, Formal Analysis, Funding acquisition, Investigation, Methodology, Project administration, Resources, Software, Supervision, Validation, Visualization, Writing – original draft, Writing – review & editing.

## Supplementary material

Supplementary Table 1 Reported gender-Associated Survival Among People with HIV-Associated Cryptococcal Meningitis.

Supplementary Figure 1 Spearman’s correlation of regulatory elements IL-10 and PD-L1 by gender among multivariate predictors of gender-specific survival among females and males who died on antifungal treatment. A-C correlation of IL-10 with A - CCL11/Eotaxin, B - CXCL10/IP-10, and C - IL-15 among females and males who died on antifungal treatment, (i - ii, respectively). D – correlation of IL-8 with IL-15 among females and males who died on antifungal treatment. E-H – correlation of PD-L1 with E – IL12p70, F – IL-15, G – CXCL10, and H – CCL11 among females and males who died on antifungal treatment (i - ii), respectively). Notable, among females and males who survived, the responses were similar and positively correlated across the panels.
