# Supplementary material for: Divergent neuroimmune signatures in the cerebrospinal fluid predict differential gender-specific survival among patients with HIV-associated cryptococcal meningitis
Source: Front Immunol. 2023 Dec 13;14:1275443. doi: 10.3389/fimmu.2023.1275443 (PMC10752005; doi:10.3389/fimmu.2023.1275443)
Supplement: Supplementary Table 1 — Reported gender-Associated Survival Among People with HIV-Associated Cryptococcal Meningitis. [file Presentation_1.pdf]

SUPPLEMENTARY MATERIALS

Supplementary Table S1. Reported gender-Associated Survival Among People with HIV-Associated Cryptococcal Meningitis.

**Article**	**Number of cases**	**Females**<br>**N (%)**	**Males**<br>**N (%)**	**Total Mortality**<br>**N (%)**	**Female Mortality**<br>**N (%)**	**Male Mortality**<br>**N (%)**
Pappas 2004 **(28)**	70	13 (18.6)	57 (81.4)			
Boulware 2010 **(29)**	170	70 (48.0)	100 (58.0)	85 (50.0) *		
Baldassarre 2014+ **(44)**	76	30 (39.5)	46 (60.5)	30 (39.5)	12 (40.0	18 (39.1)
Jarvis 2014+ **(26)**	481	231 (48.0)	250 (52.0)	163 (34.0) #	79 (34.2)	84 (33.6)
Boulware 2014 **(9)**	177	84 (47.5)	93 (52.5)	67 (37.9)		
Rajasingham 2015 **(30)**	188	100	88 (47.0)	45 (24.0) *		
Meya 2015 **(16)**	46	25 (63.0)		14 (30.0) =		
George 2017 **(31)**	158	54 (34.1)	108 (65.9)	41 (26.0) *		
Rhein 2017 **(32)**	172		113 (66.0)	69 (40.0)		
Kashef Hamadani 2018 **(33)**	55	10 (18.2)	45 (81.8)			
Meya 2019 **(34)**	3359		1522* (47.1)	949 (28.3) ^		
Pastick 2019 **(35)**	821	334 (40.7)	587 (58.7)	334 (41.3)		

Lakoh 2020 **(36)**	8	7	1	2 (25.0) [*]		
Marr 2020 **(37)**	145	50 (35.5)	95 (65.5)			
Lee 2021 **(38)**	76	28 (36.8)	48 (63.2)	10 (80.3)		
Stadelman 2021++ **(7)**	977	400 (40.9)	577 (59.1)	445 (45.5) [#]	198 (50.0)	247 (43.0)
Kalata 2021 **(39)**	678		390 (57.5)	251 (37.0) [#]		
Mansoor 2021 **(40)**	29066	6787 (23.3)	22279 (76.6)	2877 (9.9) [a]		
Deiss 2021 **(41)**	87			33 (37.9) [=]		
Zhao 2021++ **(42)**	386	83 (21.5)	303 (78.5)	58 (15.0) [a]	18 (21.7)	40 (15.2)
Jarvis 2022 **(43)**	814	323 (39.7)	491 (60.3)	218 (26.9) [#]		
Okurut (current)++	380	150 (39.5)	230 (60.5)	173 (45.5)	79 (52.7)	94 (40.9)

* - hospital deaths. = - 12 weeks deaths. # - 10 weeks deaths. ^ - 24 weeks. a – all deaths.

Studies done prior to (+) and after (++) use of optimal (delayed) initiation of ART [9].

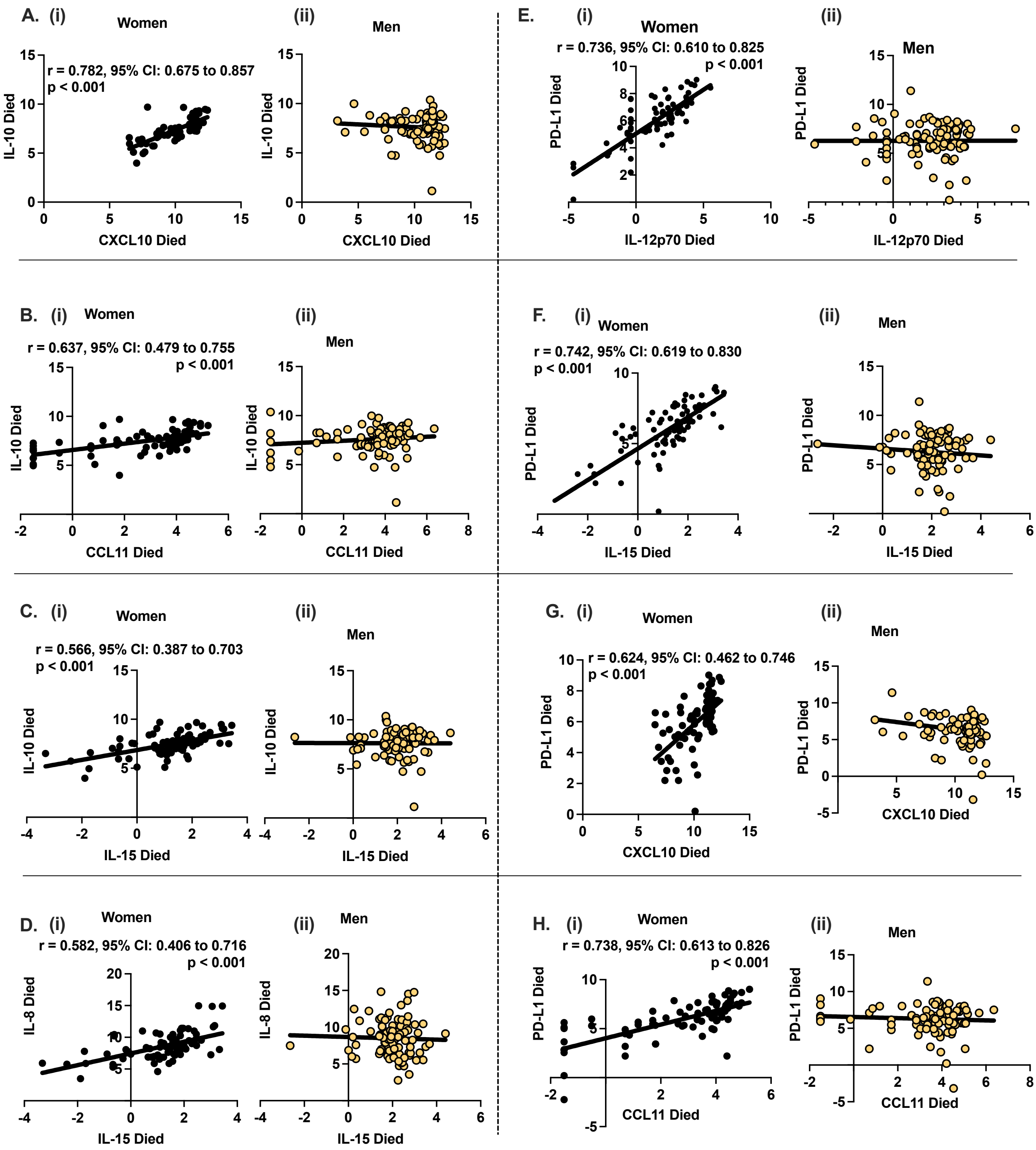

Supplementary Figure S1. Spearman's correlation of regulatory elements IL-10 and PD-L1 by gender among multivariate predictors of gender-specific survival among females and males who died on antifungal treatment. A-C correlation of IL-10 with A - CCL11/Eotaxin, B - CXCL10/IP-10, and C - IL-15 among females and males who died on antifungal treatment, (i - ii, respectively). D – correlation of IL-8 with IL-15 among females and males who died on antifungal treatment. E-H – correlation of PD-L1 with E – IL12p70, F – IL-15, G – CXCL10, and H – CCL11 among females and males who died on antifungal treatment (i - ii), respectively). Notable, among females and males who survived, the responses were similar and positively correlated across the panels.
